# Finding sequence motifs with Bayesian models incorporating positional information: an application to transcription factor binding sites

**DOI:** 10.1186/1471-2105-9-262

**Published:** 2008-06-04

**Authors:** Nak-Kyeong Kim, Kannan Tharakaraman, Leonardo Mariño-Ramírez, John L Spouge

**Affiliations:** 1 National Center for Biotechnology Information, National Library of Medicine, National Institutes of Health, Bethesda, Maryland 20894, USA

## Abstract

**Background:**

Biologically active sequence motifs often have positional preferences with respect to a genomic landmark. For example, many known transcription factor binding sites (TFBSs) occur within an interval [-300, 0] bases upstream of a transcription start site (TSS). Although some programs for identifying sequence motifs exploit positional information, most of them model it only implicitly and with *ad hoc *methods, making them unsuitable for general motif searches.

**Results:**

A-GLAM, a user-friendly computer program for identifying sequence motifs, now incorporates a Bayesian model systematically combining sequence and positional information. A-GLAM's predictions with and without positional information were compared on two human TFBS datasets, each containing sequences corresponding to the interval [-2000, 0] bases upstream of a known TSS. A rigorous statistical analysis showed that positional information significantly improved the prediction of sequence motifs, and an extensive cross-validation study showed that A-GLAM's model was robust against mild misspecification of its parameters. As expected, when sequences in the datasets were successively truncated to the intervals [-1000, 0], [-500, 0] and [-250, 0], positional information aided motif prediction less and less, but never hurt it significantly.

**Conclusion:**

Although sequence truncation is a viable strategy when searching for biologically active motifs with a positional preference, a probabilistic model (used reasonably) generally provides a superior and more robust strategy, particularly when the sequence motifs' positional preferences are not well characterized.

## Background

Transcription factor binding sites (TFBSs) provide a specific example of biologically functional sequence motifs that sometimes have positional preferences. TFBSs contribute substantially to the control of gene expression, and because of their biological importance, much experimental effort has been expended in identifying them. Because experimental identification is expensive, there are now many computational tools that identify TFBSs as the subsequences, or "motifs", common to a set of sequences. Most TFBSs correspond to short and imprecise motifs [1], however, so all computational tools in a recent contest performed rather poorly in identifying known TFBSs [2].

Although some tools have an *ad hoc *basis [3-5], other tools have a basis in the calculus of probability, and can therefore immediately and systematically combine sequence with other sources of information. Most probabilistic tools align candidate subsequences and convert the nucleotide counts in the alignment columns into a position-specific score matrix (PSSM). Most PSSMs are based on the log ratio between a motif model and a background model. Tools then identify putative motifs by maximizing the log ratio, usually with expectation maximization (EM) [6] or Gibbs sampling [7-9].

**Figure 1:**
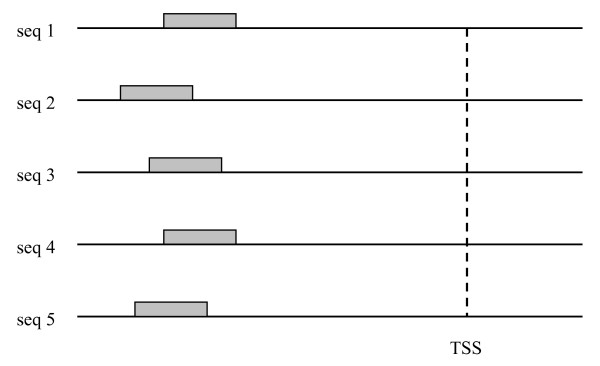
Positions of hypothetical TFBSs (gray boxes) with respect to the corresponding TSS.

Experiments have shown, however, that besides common sequence motifs, TFBSs also have positional preferences, as illustrated in Figure 1. In yeast, TFBS positions demonstrate a strong bias toward locations between 150 and 50 bases upstream of the TSS [10]. In E. coli, TFBS positions tend to be located between 400 and 0 bases upstream of the translation start site [11]. In the words of Wray *et al.*, "for at least some regulatory elements, function constrains their position with respect to the transcriptional start site" (TSS) [1]. On the other hand, the trends regarding the positional preferences of TFBSs appear inconsistent. Wray *et al. *continue "for most transcription factors, however, binding sites lack any obvious spatial restriction relative to other feature of the locus" [1].

Some computational methods do exist to exploit the positional preferences of TFBSs. The first computational study using positional preferences used an empirical prior distribution of known positional information with respect to the translation start site from the *E. coli *genome [12]. This simple method, however, is applicable only to very simple organism like *E. coli*. Another computational study used position to calculate p-values for candidate motifs that formed a cluster [13]. The p-values were based on one particular database, however, and might not generalize reliably. Moreover, the corresponding model is not a probability model, making the systematic combination of sequence and positional information problematic. Yet another computational study modeled the positional preferences of TFBSs with a uniform prior, only mentioning the possibility of a more informative prior [11]. A systematic computational study to find new TFBS motifs by exploiting positional preferences applied a chi-square test to bins of positions near TSSs [14]. The chi-square test found one 8-letter word with significant positional preferences, the "Clus1" word, TCTCGCGA. The study's use of binning probably reduced the power of statistical tests, however. Shortly thereafter, in confirmation of the reduced statistical power, a systematic study of a human promoter dataset [15] identified 801 8-letter words with a positional preferences with respect to the TSS [9]. Interestingly, although 388 of the 801 words appeared in the TRANSFAC database [16], 413 of the words did not, suggesting that TFBS positional preferences were much more pervasive than previously believed. A later study showed that in eukaryotes the distribution of TFBSs was not uniform with respect to the TSS [17]. A study using chromatin immunoprecipitation followed by DNA hybridization (ChIP-Chip experiments) inferred TFBSs within sheared DNA fragments by using prior probability distributions to model positional preference [18]. The model was not directed at identifying TFBSs by their positional preferences with respect to genomic landmarks, however. Finally, a study applied a Poisson approximation to bins of positions within promoters to identify TFBSs by their positional preferences with respect to the TSS [19].

Several studies, therefore, have examined the positional conservation of TFBSs. Consequently, TFBS positional preferences are relatively well understood, particularly when compared to most non-coding DNA. Very few computational tools systematically combine positional preference with sequence information, however, and to our knowledge, no general-purpose computational tools using positional information are currently available. Standard tools like MEME [6], AlignACE [10], and MotifSampler [20], e.g., do not use positional information. Accordingly, this article evaluates the accuracy of predictions from a Bayesian model combining sequence with positional information, implemented in the newest version of the tool A-GLAM [9]. We assessed predictions from A-GLAM with and without the positional information, using a standard dataset of sequences with known TFBSs, and were therefore able to measure the contribution of positional information to TFBS prediction accuracy.

## Results

### Results for the TSS Tompa dataset

The TSS Tompa dataset is one of two test datasets considered in this study and contains 23 data subsets (see Methods). Table 1 shows an anecdotal A-GLAM alignment using positional information for the dataset 'hm08r' from the TSS Tompa dataset, which contains 10 sequences of length 2001. Run in its ZOOPS mode (Zero Or One Per Sequence), A-GLAM returned candidate alignments with only one or zero candidate site per sequence. In addition to sequence conservation, the alignment shows positional conservation within an interval of [-220, -1], much narrower than the input interval, [-2000, 0] bp upstream of the transcription start site (TSS). The alignment also overlapped several known sites (underlined in Table 1), with a correlation coefficient of 0.574, indicating good overlap.

**Table 1:** The A-GLAM output with positional information for 'hm08r'.

Name	Start	Alignment	End	Score	E-value
seq_0	-66	GTCACGGC	-59	11.0093	6.65E-06
seq_2	-65	GTGACGTT	-58	10.3315	2.30E-05
seq_3	-58	ATGACGTC	-51	11.2688	2.94E-06
seq_5	-188	GTGACGTC	-181	11.4594	1.28E-06
seq_7	-184	CTGACGAC	-177	9.86871	4.64E-05
seq_9	-101	ATGACGTC	-94	10.9283	8.09E-06
seq_10	-220	ATCACGGC	-213	7.58906	3.78E-04
seq_11	-80	GTGACGTC	-73	11.1306	4.75E-06
seq_12	-52	CTGACGGC	-45	10.0764	3.50E-05
seq_14	-8	CTGATGTC	-1	7.60515	3.69E-04

A-GLAM predicted TFBSs in 10 data subsets in the TSS Tompa data subset hm08r'. The column "Name" shows each data subset; the column "Alignment", the corresponding predicted TFBS. The start and end positions with respect to the corresponding TSS are shown in the columns "Start" and "End". The columns "Score" and "E-value" show bit scores and E-values that A-GLAM assigned to predicted TFBSs. The known binding sites in the alignment are underlined.

Table 1 does not show the corresponding alignment without positional information, because its width was a biologically unrealistic 126 bp long. The alignment showed little positional conservation, with a range of [-2000, -1237]. It also showed essentially no overlap with the known sites, with a correlation coefficient of -0.012.

For TFBSs predicted without positional information, E-values were immoderately small, even for incorrect predictions. (Some incorrect predictions even displayed a numerical underflow E-value of 0, data not shown.) In contrast, the E-values in Table 1 were quite moderate, perhaps because they had to reconcile conflicting constraints from different sources of information on the motifs.

Alignments for more data subsets can be found in Supplementary Tables 1–6 [see Additional file 1]. We collected alignments (with positional information) whose correlation coefficient (CC) is larger than 0.08. The hm03r data subset does not appear in Tables 1–6, despite a CC of 0.386, because the corresponding alignment had a biologically unrealistic width of 224 bp. Unrealistically large alignment widths are much less common for alignments with positional information than without. In Supplementary Tables 2–6, the alignments without positional information are omitted because they show essentially no overlap with known binding sites.

Table 2 summarizes results for all 23 TSS Tompa data subsets. Some 18 out of the 23 datasets show improved predictions after adding positional information. Overall, the combined correlation coefficient (CCC; see Methods) at the bottom of Table 2 improved from -0.008 to 0.101. To evaluate the statistical significance, let *γ*_- _and *γ*_+ _denote the average correlation coefficient for each data subset without and with positional information. A one-sample Wilcoxon test against the one-sided null hypothesis *γ*_- _≥ *γ*_+ _yielded a p-value of 0.002, supporting the alternative hypothesis that *γ*_- _<*γ*_+_.

**Table 2:** The correlation coefficients for the TSS Tompa data subsets

Data Subset	Without positional information	With positional information	Improvement
hm01r	-0.012	-0.007	0.005
hm02r	-0.009	-0.007	0.002
hm03r	-0.037	0.386	0.423
hm04r	-0.008	-0.005	0.003
hm05r	-0.031	-0.019	0.012
hm06r	-0.014	0.156	0.170
hm07r	-0.015	-0.015	-0.001
hm08r	-0.012	0.574	0.586
hm09r	-0.011	0.358	0.369
hm10r	-0.019	0.083	0.102
hm11r	-0.028	-0.012	0.016
hm13r	-0.015	-0.016	-0.001
hm14r	0.204	-0.018	-0.222
hm15r	-0.011	-0.012	-0.002
hm16r	-0.011	-0.006	0.005
hm17r	-0.015	-0.012	0.004
hm18r	-0.018	0.094	0.112
hm19r	-0.010	-0.007	0.003
hm20r	-0.026	0.046	0.073
hm21r	0.401	0.384	-0.016
hm22r	-0.020	-0.020	0.000
hm24r	-0.016	-0.010	0.006
hm26r	-0.016	0.099	0.115

Combined CC	-0.008	0.101	0.109

Table 2 shows the correlation coefficients for A-GLAM's predictions on the 23 subsets of the TSS Tompa dataset. The column, "Improvement", quantifies the effect of positional information on predictions, by showing the difference between the correlation coefficients in the second and third columns, "Without Positional Information" and "With Positional Information".

### Results for TRANSFAC dataset

The TRANSFAC dataset contains 82 data subsets. Supplementary Table 8 contains detailed results for the input interval of [-2000, 0]. With the addition of positional information, the CCC has improved from -0.009 to 0.027 with a p-value of 10^-8 ^(Wilcoxon test as above). The CCC for TRANSFAC dataset (0.027) is smaller than for TSS Tompa dataset (0.101), and the positional information makes a more significant change in the CCC for the TRANSFAC dataset (p = 10^-8^) than for the TSS Tompa dataset (p = 0.002), probably because the TRANSFAC dataset contains 82 data subsets; the TSS Tompa dataset, only 23. In the case of subtle differences, the larger TRANSFAC dataset provides more evidence, leading to smaller p-values.

### Cross-validation using TSS Tompa dataset

Because we used known binding sites to estimate the hyperparameters of the model (see Methods), one might suspect over-fitting. Moreover, because the distribution of locations might vary from one type of TFBS to another, the proposed model might not be appropriate for the discovery of unknown binding sites of different types of TFBSs. Cross-validation addressed these issues (see Methods).

**Figure 2:**
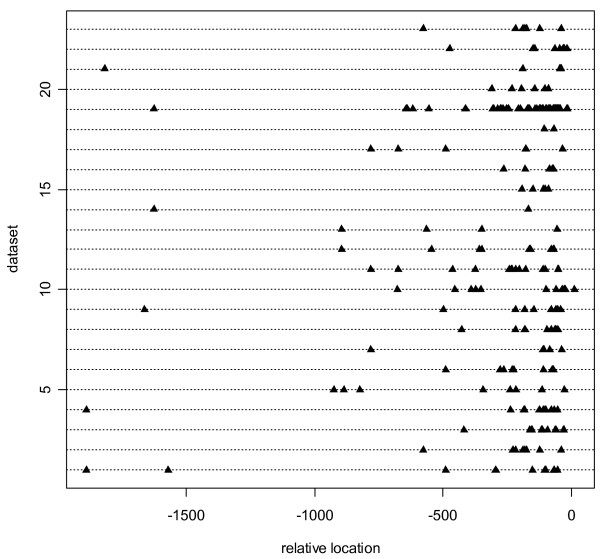
**Distribution of known locations of binding site in TSS Tompa dataset**. The x-axis is anchored on the TSS, denoted as location 0. All sequences in each test subset are collapsed into a single line; hence the 23 data subsets are shown as 23 different horizontal lines. Each data subset contains TFBSs corresponding to a single specific transcription factor.

Over the 100 random partitions from TSS Tompa dataset, the sample average of the CCC was 0.086; its sample standard deviation, 0.027; its 90% confidence interval, (0.049, 0.133); and its range, (0.029, 0.155). (The TRANSFAC dataset was not used for 5-fold cross-validation because of amount of computation required.) The CCC for the model using sequence information alone was -0.008. Because the CCC for sequence alone lay outside the range (0.029, 0.155) of the 100 CCCs using positional information in the 5-fold cross-validation, positional information improved prediction accuracy significantly. The actual CCC for the model using both sequence and positional information was 0.101 (see Table 2), well within the 90% confidence interval from cross-validation. The different types of known sites have quite diverse distributions (see Fig. 2), so we expect occasional misspecification of hyperparameters **η **in our model (see Methods). The 5-fold cross-validation shows, however, that classification accuracy is not excessively sensitive to the hyperparameter estimation or, by extension, to the locations of the known sites.

### Truncation effect on sequences of test datasets

**Figure 3:**
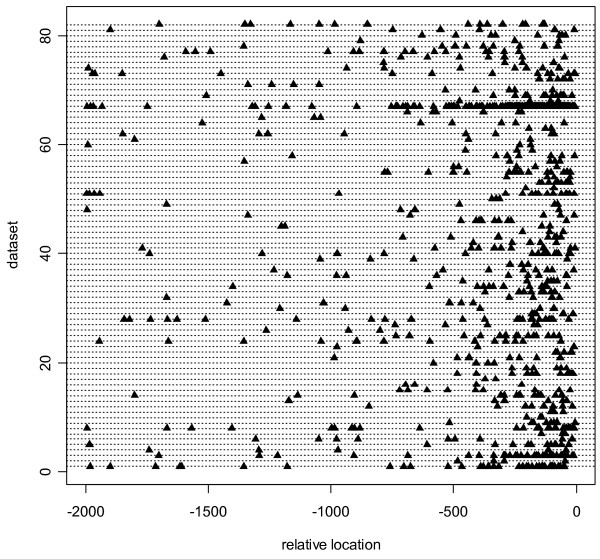
**Distribution of known locations of binding site in TRANSFAC dataset**. The x-axis is anchored on the TSS, denoted as location 0. All sequences in each test subset are collapsed into a single line; hence the 82 data subsets are shown as 82 different horizontal lines. Each data subset contains TFBSs corresponding to a single specific transcription factor.

Figures 2 and 3 suggest that a truncated input sequence interval of, say, [-500, 0] or [-250, 0] might incorporate positional information as well as a Bayesian positional model applied to the full interval [-2000, 0]. Accordingly, in addition to the full interval [-2000, 0], we tested 3 truncated intervals [-1000, 0], [-500, 0], and [-250, 0]. (See Supplementary Table 7 and 8 for details.) The predictive accuracy, as represented by the CCCs in Table 3, indicate that truncation on its own, without any Bayesian positional modeling, improved the motif predictions. Moreover, predictive improvements due to modeling position gradually disappeared as the truncation reduced the interval to [-250, 0]. Note, however, that positional modeling never significantly hurt the predictive accuracy, even with truncated input sequences.

**Table 3:** The effect of truncating the sequence upstream of the TSS

Sequence range	TSS Tompa Dataset			TRANSFAC Dataset		
	
	Without positional info	With positional info	p-value	Without positional info	With positional info	p-value
[-2000, 0]	-0.008	0.101	0.002	-0.009	0.027	10^-8^
[-1000, 0]	0.086	0.098	0.583	0.050	0.066	0.112
[-500, 0]	0.125	0.133	0.338	0.077	0.078	0.070
[-250, 0]	0.139	0.139	0.054	0.094	0.076	0.603

The first column shows the sequence range upstream of the TSS given as input to A-GLAM. The change of CCC from modes with and without positional information for the TSS Tompa and TRANSFAC datasets is displayed in the corresponding groups of three columns. The third column of each group shows a Wilcoxon p-value, which evaluates the difference between the CCCs in the previous two columns. Because not all TFBSs in our datasets are known, small improvements in the CCC correspond to true improvements of unknown magnitude. In particular, e.g., in the Table, two CCC values rounded to 0.139 have unseen decimals different enough to have a p-value of 0.054. To view results for individual sites in the Tompa dataset, see Supplementary Table 7 [see Additional file 1].

## Discussion

The new version of the A-GLAM program ('anchored gapless local alignment of multiple sequences', written in C++) [9,21] can incorporate positional information by implementing the model from the Methods section in a Gibbs sampler. A-GLAM already has several desirable features when predicting transcription factor binding sites (TFBSs). First, it optimizes motif width automatically, without user input. Second, it reports theoretically accurate E-values for candidate TFBSs. Finally, it implements a theoretically sound context-dependent Markov background model, which yielded better predictions than different, *ad hoc *Markov background models or the conventional background model of independent bases [22]. With its Markov background model, a rigorous statistical evaluation showed that even before the addition of positional information, A-GLAM's predictive accuracy was competitive with any state-of-the-art motif-finding tool [22].

At the outset, we point out that all motif-finding tools have had notorious difficultly with the original Tompa dataset [2]. Our TSS Tompa test dataset is even more difficult than the original Tompa dataset. Its data subsets often contained fewer sequences than the corresponding original Tompa subset. Moreover, our sequences were on average longer than the corresponding original Tompa sequence. Thus, conventional motif-finding tools should perform more weakly on our TSS Tompa test dataset than on the original Tompa dataset.

The Bayesian model in this paper combines sequence and positional information to predict putative TFBSs. Its implementation in A-GLAM permits users either to accept our default hyperparameters **η **for the prior distribution or to select their own. Although complete flexibility in the selection of hyperparameters can permit inappropriate or excessively aggressive choices, extensive cross-validation showed that the usual priors place mild restrictions on the predictions, so the model is very robust against misspecification of its hyperparameters or, by extension, to the locations of known sites. In other words, the prior does not dictate the alignment; instead, it loosely guides the alignment and permits the data to "speak for themselves". If motifs do not cluster by position, A-GLAM might therefore still find motifs sharing sequence but not position. We therefore make the following recommendation to users: in the absence of a strong reason to the contrary, they should accept A-GLAM's default hyperparameters.

To use positional information to find biologically active sites, A-GLAM's positional model requires the input sequences to be anchored on a genomic landmark, e.g., to find TFBSs, the model might be anchored to TSSs. Because a single gene might correspond to several alternative TSSs [23], however, TSS multiplicity might initially appear to cause problems. Moreover, the TSS itself can have either "sharp" or "broad" positional preference within a promoter [24]. Variability of the TSS position within a promoter reduces the positional information available to A-GLAM, possibly explaining the uneven improvement in prediction across our data subsets. A-GLAM's statistical model examines sequence as well as positional information, however, so it retains robustness against a mild misspecification of the TSS, say, within a few hundred bases of the true position, so alternative TSSs or TSSs with a typical broad positional distribution are unlikely to degrade predictions seriously when positional information is used. A-GLAM's users should note, however, if a TSS is specified, e.g., a kilobase away from the relevant position, positional information might severely distract A-GLAM from finding the desired TFBSs. On the other hand, however, different positions relative to the TSS containing exactly the same sequence have long been known to be associated with different TFBS biological functions [25]; in other cases, they might also be associated with alternative TSSs or TSSs with a broad positional distribution. Up to now, because computational studies of positional control of transcription have had to rely on *ad hoc *methods, A-GLAM now has a unique potential among general motif-prediction tools. Even if two functionally different sets of TFBSs have similar motifs, A-GLAM can differentiate them by position alone and report the two sets separately. It would be very interesting if someone using A-GLAM identified two sets of TFBSs of similar sequence corresponding to two different functionalities or TSSs.

The sequences in our study used the upstream positions from -2000 to 0 bp relative to the TSS to evaluate A-GLAM's accuracy in predicting TSSs. Because our purpose in this article was to evaluate A-GLAM's ability to find biologically active sequence motifs in general, there is no scientific reason not to use the 3' UTR region as a "genomic anchor" to identify nearby regulatory elements. A similar statement applies to any set of regulatory elements (e.g., TFBSs, miRNA binding sites, etc.) around any genomic landmark (e.g., the TSS, the 3' UTR, etc.).

Indeed, if its main purpose was not evaluation of the predictive accuracy of A-GLAM's positional model, this article could have restricted its input sequences to intervals downstream of the TSS, e.g., [0, 1000] bp instead. With the TSS still providing the genomic anchor, A-GLAM could have searched for motifs associated with, e.g., 5' UTRs or translation start sites, which are usually within a few hundred base pairs downstream of a TSS. Thus, positional restrictions on the input sequence could focus A-GLAM's search on sequence motifs with different biological functions.

In practice, however, restricting the input interval requires great care. Unlike the TFBSs in our test datasets, many sequence motifs have poorly characterized distributions. On one hand, excessively stringent truncation of the input interval to, say, [-125, 0] would probably have removed many TFBSs from consideration in our study. On the other hand, positional modeling generally improved the accuracy of motif prediction, never hurting it significantly, even when input sequences were truncated. In the search for novel sequence motifs, therefore, we recommend that the use of Bayesian positional modeling on an input sequence whose length is generous (but not too generous) relative to the locations of known motifs.

Since the previous study showed that A-GLAM is one of the top performers among existing tools for *de novo *TFBS discovery [22], we believe that A-GLAM now easily outperforms its competitors whenever positional information is available and relevant. "Positional genomics" exploits the information provided by genomic landmarks (like the TSS), yielding a "poor man's alignment", even when the precise sequence alignments are unavailable. Given the power of comparative genomics, which depends on accurate alignments, positional genomics presents many interesting possibilities.

## Conclusion

We proposed a Bayesian model for incorporating positional preference of TFBS with respect to a genomic landmark, e.g., a TSS. The results on our test datasets show that a positional model can produce statistically significant improvements in the accuracy of motif prediction. Our cross-validation study shows that the prior distribution of our positional model is robust against mild misspecification of its parameters. Our study of truncated input sequences indicates that the positional model provides a superior and more robust strategy than sequence truncation, especially when the positional preferences of sequence motifs are not well characterized.

## Availability

The A-GLAM program and all datasets relevant to this article can be found online [26].

Project name: A-GLAM 2.1

Project home page: 

Operating system: Linux

Programming language: C++

Licence: No license required.

## Methods

### The two test datasets

Our first test dataset was a subset of the "real" human sequences in the "original Tompa dataset", from [2]. The original Tompa dataset does not annotate any experimentally verified TSS positions, which were supplied from the Database of Transcription Start Sites (DBTSS) [27], as follows. BLAT [28] searched the DBTSS for hits to sequences in the original Tompa dataset. The DBTSS is incomplete, so when BLAT returned no hits in a sequence, the corresponding sequence was discarded. After the BLAT search, the dataset contained 26 data subsets, each composed of human sequences with a known TSS, and each corresponding to a single type of TFBS, like the original Tompa data subsets. We then discarded data subsets with 0 or 1 sequences, resulting in our "TSS Tompa dataset", which contained 23 data subsets. Each data subset contained from 2 to 26 sequences, and each sequence contained any number of known TFBSs, including 0. To encompass systematically all known TFBSs in the sequences, each sequence was expanded to contain proximal promoter regions from -2000 to 0 bp (upstream) relative to the corresponding TSS.

Our second test dataset was constructed from: (1) the latest human genome build (NCBI Build 36, ); (2) transcriptional start sites (TSS) from the database of transcription start sites (DBTSS) [27]; and (3) experimentally characterized TFBSs from the TRANSFAC database (professional version 11.2) [29]. Briefly, TSS and TRANSFAC sites were mapped to the human genome using MegaBLAST [30], yielding a set of proximal promoter DNA sequences [15,31] annotated with experimentally characterized TSSs and TFBSs. In this paper, the resulting sequences are called our "TRANSFAC dataset". The TRANSFAC dataset contains 82 data subsets, each subset containing 2 to 101 sequences, and each sequence containing at least one instance of known TFBSs. Like the TSS Tompa data subsets, each data subset corresponded to a single type of TFBS. Like our TSS Tompa dataset, the range of TRANSFAC dataset is from -2000 to 0 bp (upstream) relative to the corresponding TSS.

A standard measure of prediction accuracy, the correlation coefficient, described elsewhere [22], evaluated TFBS predictions within our test dataset.

### A Bayesian model for positional preferences

Our model for TFBSs uses two sources of information: sequence and position. We discuss sequence later, to focus on the novelties of position first.

Figure 2 displays the positions of all known TFBSs within the data subsets of the TSS Tompa dataset. Figure 2 collapses all sequences in each test subset into a single line anchored at the TSS. Thus, the 23 lines represent the 23 data subsets. Figure 2 shows that the TFBSs in several data subsets display positional preferences with respect to the TSS. Many TFBSs are upstream of the TSS, possibly clustered around certain positions. Accordingly, we search for TFBS positions that are normally distributed, with unknown center and dispersion, near the TSS. (Mathematical convenience facilitates the choice of the normal distribution.) Analogous to Figure 2, Figure 3 contains the positions of all known TFBSs in the TRANSFAC dataset. The TRANSFAC dataset displays the same basic distributional characteristics as the TSS Tompa dataset in Figure 2.

Fix a data subset in Figure 2 or 3, and assume it contains some number *n *of unknown TFBSs with locations *x*_1_,...,*x*_*n *_relative to the TSS. For later reference, let ${\bar{x}}_{n}=n^{-1}\sum_{i=1}^{n}x_{i}$ and $s_{n}^{2}=n^{-1}\sum_{i=1}^{n}{\left(x_{i}-{\bar{x}}_{n}\right)}^{2}$ be the sample mean and sample standard deviation. Assume **x **= (*x*_1_,...,*x*_*n*_) constitute independent samples from a Normal (*μ*, *λ*) distribution, with mean *μ *and reciprocal variance (also known as "precision") *λ *= 1/*σ*^2^. Given the normal parameters **θ **= (*μ*, *λ*), the positions **x **have the likelihood function

(1) $$p(x|\theta )={\left(\frac{\lambda }{2\pi }\right)}^{n/2}\exp\left\{-\frac{1}{2}\lambda \sum_{i=1}^{n}{\left(x_{i}-\mu \right)}^{2}\right\}.$$

Parenthetically, to avoid confusion, the sequence locations *x*_1_,...,*x*_*n *_are integers, but the use of continuous distributions (e.g., the normal) as approximations simplifies the algebra enormously. Similarly, the locations *x*_1_,...,*x*_*n *_might be confined to a finite interval (e.g., they might be within a finite piece of DNA). The seemingly unrestricted normal distribution remains appropriate, however, because its rapidly vanishing squared exponential form (as in Eq) effectively confines its samples to a finite interval.

Now, let the normal parameters **θ **= (*μ*, *λ*) have a uniform-gamma prior distribution, in which *μ *and *λ *have independent prior distributions. The prior for *μ *is the continuous Uniform [*a, b*] distribution on some closed interval [*a, b*] (*a *<*b*), with constant density *p*(*μ*) = (*b *- *a*)^-1 ^for *μ *∈ [*a*, *b*]. The prior for *λ *is a Gamma(*α*, *β*) distribution with parameters *α*, *β *> 0, with density

$$p(\lambda )=\frac{\beta^{\alpha }}{\Gamma (\alpha )}\lambda^{\alpha -1}\exp(-\beta \lambda )$$

for *λ *≥ 0. The uniform-gamma prior distribution for **θ **= (*μ*, *λ*) therefore has the joint density function

$$p(\theta )={\left(b-a\right)}^{-1}\cdot \frac{\beta^{\alpha }}{\Gamma (\alpha )}\lambda^{\alpha -1}\exp(-\beta \lambda ),$$

for *μ *∈ [*a*, *b*] and *λ *≥ 0.

Practical suggestions for the numerical values of *α *and *β *are given below.

Our aim is to provide a figure of merit for Gibbs sampling based on the predictive distribution *p*(**x**) = ∫*p*(**x**|**θ**)*p*(**θ**)*d***∫ **of the locations **x**. Gibbs sampling conditions on the locations **x **= (*x*_1_,...,*x*_*n*_) to determine the conditional predictive distribution of the location *x*_*n*+1 _= *x *of another TFBS (see Eq (2) below). After extensive algebraic manipulation of the relevant integrals, the conditional predictive distribution is

(2) p(x|x)=p(x,x)p(x)=Γ(12(ν+1))Γ(12ν)(νπ)−1/2σ−1{1+(x−x¯n)2νσ2}−12(ν+1),

a Student t-distribution whose parameters are $\nu =2\left[\alpha +\frac{1}{2}(n-1)\right]$, ${\bar{x}}_{n}$, and

$$\sigma^{2}+\frac{n+1}{n}\frac{\beta +\frac{1}{2}ns_{n}^{2}}{\alpha +\frac{1}{2}(n-1)}.$$

The t-distribution has mean ${\bar{x}}_{n}$ for *v *> 1 and variance *v*(*v *-2)^-1^*σ*^2 ^for *v *> 2.

The result in Eq (2) ignores the restriction *μ *∈ [*a*, *b*]. If [*a*, *b*] covers most of the range [*a'*, *b'*] of the locations **x **(e.g., *a*-3*σ *<*a' *<*b' *<*b*+3*σ*), then analysis will confirm that under appropriate mathematical hypotheses, Eq (2) approximates the desired conditional predictive distribution accurately.

The prior distribution is fully specified by a list of the hyperparameters *a*, *b*, *α*, and *β*. As indicated above, any sufficiently generous interval [*a*, *b*] containing the locations **x **suffices for present purposes. The input sequence range (e.g., in the case of TSS Tompa's dataset as well as TRANSFAC dataset, from -2000 to 0 bp relative to the corresponding TSS) is a practical choice for [*a*, *b*]. In contrast, the selection of *α *and *β *can be delicate. On one hand, a user can provide subjective preferences for *α *and *β*, yielding a precision *λ *with mean *αβ*^-1 ^and variance *αβ*^-2^. On the other hand, *α *and *β *can be estimated from the distributions of experimentally verified TFBSs, as follows.

Suppose we have *k *data subsets, where the *i*-th data subset (*i *= 1,...,*k*) yields a known vector **x**_*i *_of locations for a particular TFBS. Each data subset **x**_*i *_corresponds to a different set of hyperparameters {*θ*_*i *_= (*μ*_*i*_, *λ*_*i*_)}_*i *= 1,...,*k *_chosen from a common uniform-gamma prior with unknown parameters **η **= (*α*, *β*). The predictive distribution of the data is

$$\begin{array}{c} p(x_{1},...,x_{k}|\eta )=\left\{\int p(x_{1}|\theta_{1})p(\theta_{1}|\eta )d\theta_{1}\right\}\\ \ldots \left\{\int p(x_{k}|\theta_{k})p(\theta_{k}|\eta )d\theta_{k}\right\} \end{array}.$$

Maximization of the predictive distribution yields the so-called type-II maximum likelihood estimate for **η **= (*α*, *β*) [32].

In this study, based on our two datasets, the type-II maximum likelihood estimate of *α *and *β *were selected. The value of *α *was 0.8424; of *β*, 25790 for TSS Tompa dataset; *α*, 0.5825, *β*, 12818, for TRANSFAC dataset. Thus, the distribution of the precision *λ *had mean 3.27 × 10^-5 ^(4.54 × 10^-5^, for TRANSFAC dataset), giving the scale parameter *σ *= *λ*^-1/2 ^an approximate mean 175 (148, for TRANSFAC dataset). (The lengths of typical input sequences are several hundreds to a couple of thousand, e.g., in our dataset, the lengths are all 2000.) Now, 95% of the realizations from a Normal (*μ*, *λ*) distribution fall into the interval (*μ*-2*σ*, *μ*+2*σ*) of length 4*σ*. Because 4*σ *= 4(175) = 700 (592, for TRANSFAC dataset), the above selection of *α *and *β *makes the prior distribution quite broad, permitting the data "to speak for themselves".

### Some comments on the distributional choices for the prior and likelihood

The normal distribution might be challenged as an inappropriate form for the likelihood. In most of the data subsets in Figure 2 or3, it is completely justifiable, but does appear untenable for a few. Although mathematical convenience facilitates the choice of a normal distribution, one could propose alternative distributional forms, usually at the expense of greater complexity. The normal distribution is quite adequate, however, when modeling any cluster lacking distant "orphan" locations.

Similarly, a uniform prior for the normal mean *μ *might be challenged. In fact, we implemented the same model with a normal-chi-square prior for **θ **= (*μ*, *λ*). In our hands, both models produced comparable results on our test dataset (data not shown).

### Gibbs sampling using both sequence and position

As noted above, Gibbs sampling requires only conditional predictive distributions. Because of the uniform prior for *μ*, multiplying the conditional predictive distribution in Eq (2) by (an ultimately irrelevant factor of) (*b *- *a*) yields an approximation for the conditional predictive odds ratio with respect to the uniform background model. Taking logarithms and adding subscripts for "location", yields a log-odds score Δ*s*_ [*l*] _(*x*_ [*l*] _| **x**_ [*l*]_) for location.

Now, consider the sequence information. Let the *n *locations **x**_ [*l*] _initiate subsequences **x**_ [*s*] _of length *w *(for "window"). Let the count of nucleotide *j *in the *i*-th column of the window be *c*_*i*,*j*_, so the total count in each position is *c *= ∑_(*j*)_*c*_*i,j *_= *n*. As in the conditional predictive distribution above, add another subsequence **x**_ [*s*] _of length *w *to the data. Let *δ*[*i*, *j*] equal 1 if the new subsequence contains nucleotide *j *in its *i*-th position, and 0 otherwise. Our previous work [9] postulated a familiar model [7,8], that the TFBS sequences follow a multinomial motif model with a Dirichlet prior. In the prior, the nucleotide pseudo-counts were {*a*_*j*_} (*a *= ∑_(*j*)_*a*_*j*_). The background model was the so-called "independent letters model" with probabilities {*p*_*j*_}. Effectively, our previous work gave the conditional log-odds ratio of the subsequence *x*_ [*s*]_, given the subsequences **x**_ [*s*]_, as

(3) $$\Delta s_{\left[s\right]}(x_{\left[s\right]}|x_{\left[s\right]})=\sum_{i=1}^{w}\sum_{j=1}^{4}\delta [i,j]\log\left[\left(\frac{c_{ij}+a_{j}}{c+a}\right)/p_{j}\right].$$

If sequence and position are independent in both the motif and background models, the corresponding conditional predictive log-odds ratio is Δ*s*(*x *| **x**) = Δ*s *_[*s*] _= (*x *_[*s*] _| *x *_[*s*]_) + Δ*s *_[*l*] _(*x *_[*l*] _| **x **_[*1*]_). Conditional predictive log-odds ratios can be added to generate the log-odds ratios for any dataset **x **step by step. Thus, Eqs (2) and (3) completely specify a predictive log-odds ratios for use as the figure of merit in Gibbs sampling. The present article actually replaces the independent letters model for the sequence background with a Markov model of order 3 [22], but the principles are the same.

Having established the separate roles of sequence and location, we drop the subscripts [*s*] and [*l*] below, particularly in *x*_*i*_, which now represents the sequence and location of the *i*-th candidate TFBS.

### A p-value for each candidate TFBS

For consistency with other computer programs (and because it makes little practical difference), to calculate a p-value for the *i*-th candidate TFBS *x*_*i*_, we consider the self-predictive score Δ*s*(*x*_*i *_| **x**), where ***x ***= (*x*_*i*_, ..., *x*_*i*_, *x*_*n*_) includes *x*_*i*_. Because sequence and location are independent variates in both the motif and background models, the distribution of Δ*s*(*x*_*i *_|**x**) is a convolution, i.e.,

$$\begin{array}{c} \mathbb{P}\left\{\Delta s(x_{i}|x)\geq t\right\}=\sum_{\left(r\right)}\mathbb{P}\left\{\Delta s_{\left[s\right]}(x_{i,[s]}|x_{\left[s\right]})=r\right\}\\ \mathbb{P}\left\{\Delta s_{\left[l\right]}(x_{i,[l]}|x_{\left[l\right]})\geq t-r\right\} \end{array}.$$

Existing methods [33,34] determine the distribution of Δ*s*_ [*s*]_, and the distribution of Δ*s*_ [*l*] _is known. Thus, a p-value can be assigned to each candidate site.

### *k*-fold cross-validation for sensitivity of hyperparameter selection

The *k*-fold cross-validation method estimates error rates in classification problems accurately [35]. The *k*-fold cross-validation splits the available data containing known classification labels into *k *mutually exclusive "partitions", so that each partition contains about the same amount of data. It then sets aside one of *k *partitions as the test set, and uses remaining *k *- 1 partitions as a training set to estimate the statistical parameters underlying the classification rule. After repeating the estimation process *k *times, leaving out each partition in turn, the average of the resulting classification errors estimates the error rate of the rule. The choice of 5 or 10 for *k *generally overcomes the effects of replicated data, which would otherwise render the test and training data unduly similar [35]. In the present context, known sites provide estimates of the hyperparameters *η *= (*α*, *β*). In our study, cross-validation with *k *= 5 partitions was most appropriate to address over-fitting, because we have only 23 different datasets in TSS Tompa dataset. To illustrate the 5-fold cross-validation, consider the partition 23 = 5 + 5 + 5 + 4 + 4. First, set aside the first "5" of the 23 data subsets as the test set **x**_1_, and estimate the hyperparameters **η **by maximizing the value of *p*(**x**_2_,...,**x**_5_|**η**), where **x**_2_,...,**x**_5 _are the 18 = 5 + 5 + 4 + 4 training sets. With the estimated hyperparameters **η**, A-GLAM then makes predictions on the test set **x**_1_. The 5-fold cross-validation then repeats the procedure, taking each of the partitions **x**_2_,...,**x**_5 _in turn as the test set.

To eliminate the results' dependence on the partition, the partition was chosen randomly 100 times, and the results averaged.

### A-GLAM Settings for the Test Predictions

To compare the model with positional information and the model without positional information (i.e., using sequence alone), we ran A-GLAM in the ZOOPS (Zero or One Occurrence Per Sequence) mode, where A-GLAM reports zero or one instance of the motif element for each sequence. Somewhat arbitrarily, we restricted the search space to the strands in the test dataset, without the complementary strands.

## Authors' contributions

N–KK proposed the Bayesian model, implemented it in A-GLAM, and ran the program on the test datasets; KT and LM–R generated the test datasets and extracted the transcription start site information; JLS conceived and supervised the study.

## Supplementary Material

Additional file 1 Additional alignments for the TSS Tompa dataset and the complete data corresponding to the summary in Table 3. Supplementary Tables 1–6 contain additional alignments for the TSS Tompa dataset. Supplementary Table 7 summarizes truncation effects for the TSS Tompa dataset; Supplementary Table 8, for the TRANSFAC dataset. Click here for file
