# Supplementary material for: Finding sequence motifs with Bayesian models incorporating positional information: an application to transcription factor binding sites
Source: BMC Bioinformatics. 2008 Jun 4;9:262. doi: 10.1186/1471-2105-9-262 (PMC2432075; doi:10.1186/1471-2105-9-262)
Supplement: Additional file 1 — Additional alignments for the TSS Tompa dataset and the complete data corresponding to the summary in Table 3. Supplementary Tables 1–6 contain additional alignments for the TSS Tompa dataset. Supplementary Table 7 summarizes truncation effects for the TSS Tompa dataset; Supplementary Table 8, for the TRANSFAC dataset. [file 1471-2105-9-262-S1.doc]

Supplementary Table 1. Alignments for hm21r in PPR Tompa dataset.

(a) Alignment with positional information (CC=0.384)

| Name | Start | Alignment | End | Score | E-value |
| --- | --- | --- | --- | --- | --- |
| seq_1 | -239 | CTCCCTATTTGGCCATCCCCCTGA | -216 | 20.5922 | 3.59E-11 |
| seq_2 | -226 | CTCCATATACGGCCCGGCCCGCGT | -203 | 21.3477 | 8.40E-12 |
| seq_3 | -316 | GTCCATATTAGGACATCTGCGTCA | -293 | 19.0769 | 5.00E-10 |
| seq_4 | -98 | ATCCTTTTATGGCCCTGTCCCTAT | -75 | 18.7977 | 7.92E-10 |

(b) Alignment without positional information (CC=0.401)

| Name | Start | Alignment | End | Score | E-value |
| --- | --- | --- | --- | --- | --- |
| seq_1 | -238 | TCCCTATTTGGCCATCCCCCT | -218 | 16.7866 | 2.28E-06 |
| seq_2 | -225 | TCCATATACGGCCCGGCCCGC | -205 | 17.6227 | 5.76E-07 |
| seq_3 | -315 | TCCATATTAGGACATCTGCGT | -295 | 17.811 | 4.02E-07 |
| seq_4 | -97 | TCCTTTTATGGCCCTGTCCCT | -77 | 16.3436 | 5.12E-06 |

Note: Known binding sites are underlined in the alignment. Considering a minor difference at the boundary region, these two alignments are essentially equivalent. These results show that using positional information does not hurt A-GLAM’s performance at least for this data subset where the alignment without positional information already performed well.

Supplementary Table 2. Alignment with positional information for hm09r in PPR Tompa dataset (CC=0.358).

| Name | Start | Alignment | End | Score | E-value |
| --- | --- | --- | --- | --- | --- |
| seq_0 | -65 | GTGACGT | -59 | 11.5857 | 9.00E-07 |
| seq_1 | -153 | CTGACGG | -147 | 8.73465 | 1.16E-04 |
| seq_2 | -49 | GTGACGT | -43 | 10.6198 | 1.10E-05 |
| seq_3 | -188 | GTGACGT | -182 | 11.5714 | 9.19E-07 |
| seq_5 | -71 | CTGGCGT | -65 | 8.69853 | 1.21E-04 |
| seq_7 | -184 | GTCACGG | -178 | 8.18013 | 2.20E-04 |
| seq_8 | -161 | GTGAACT | -155 | 7.10153 | 6.73E-04 |
| seq_9 | -80 | GTGACGT | -74 | 10.2681 | 1.76E-05 |
| seq_12 | -202 | GTGACGT | -196 | 10.4218 | 1.45E-05 |
| seq_14 | -254 | CTGGCGT | -248 | 7.72574 | 3.88E-04 |

Note: Alignment without positional information demonstrated poor performance (CC=–0.011), implying virtually no overlap with the known binding sites (data not shown).

Supplementary Table 3. Alignment with positional information for hm06r in PPR Tompa dataset (CC=0.156).

| Name | Start | Alignment | End | Score | E-value |
| --- | --- | --- | --- | --- | --- |
| seq_0 | -117 | CGGTCACGTGCCCAGAACGTCCGGCGTTCGCCCCG | -83 | 28.3733 | 1.70E-15 |
| seq_1 | -76 | CCCTCCCCGCCCCAGCGGCGCATGCGCCGCGCTCG | -42 | 21.1767 | 1.69E-10 |
| seq_2 | -84 | CCGTCACGTGGCCAGAAGCTGGCCAATCCGGTTTG | -50 | 27.4813 | 9.39E-15 |
| seq_3 | -165 | GCGTCCCGGCGCTAGGAGGGACGCACCCAGGCCTG | -131 | 23.2843 | 9.21E-12 |
| seq_4 | -374 | GCGGGCCGGGGCCGGGGCCTGCGGCGTCGTGCGCG | -340 | 24.5161 | 1.44E-12 |

Note: Alignment without positional information demonstrated poor performance (CC=–0.014), implying virtually no overlap with the known binding sites (data not shown).

Supplementary Table 4. Alignment with positional information for hm26r in PPR Tompa dataset (CC=0.099).

| Name | Start | Alignment | End | Score | E-value |
| --- | --- | --- | --- | --- | --- |
| seq_0 |  | absent |  |  |  |
| seq_1 | -225 | GCGCGAGGGGCGGGGAGCGGGAGGCGGGGTCGGACCTGCACGCCC | -181 | 27.8155 | 2.52E-13 |
| seq_2 | -57 | GCCGGGAGCGGGGCGAGCGGGCGGGGGTTGTCAGTCCGATCTCGC | -13 | 32.7129 | 1.23E-16 |
| seq_3 | -156 | GCCCCGCCCCTCTCGAACGCCTTCGCGCGATCGCCCTGGAAACGC | -112 | 27.8168 | 2.52E-13 |
| seq_7 | -152 | GCGCCCGGCCCAGTGCGCGCGGCCGGGTGTTTCGCCTGGAGCCGC | -108 | 30.0614 | 9.38E-15 |
| seq_8 | -134 | GCGGGGGGAGCGGCGCGCGGGTGCTGGGGGACCGACCCCTCCCGC | -90 | 29.6066 | 1.91E-14 |

Note: Alignment without positional information demonstrated poor performance (CC=–0.016), implying virtually no overlap with the known binding sites (data not shown).

Supplementary Table 5. Alignment with positional information for hm18r in PPR Tompa dataset (CC=0.094).

| Name | Start | Alignment | End | Score | E-value |
| --- | --- | --- | --- | --- | --- |
| seq_0 | -648 | CAACCTGGTAAAAATAGTAAGA | -627 | 16.8633 | 8.23E-09 |
| seq_1 | -853 | CAGATAGCTGTGCATACATAAT | -832 | 14.9061 | 1.19E-07 |
| seq_2 | -692 | CAAATAGGTACGGATAAGTAGA | -671 | 22.4711 | 3.78E-13 |
| seq_3 | -679 | CAAACTAGTACAGATAGTTACT | -658 | 20.2435 | 3.40E-11 |

Note: Alignment without positional information demonstrated poor performance (CC=–0.018), implying virtually no overlap with the known binding sites (data not shown).

Supplementary Table 6. Alignment with positional information for hm10r in PPR Tompa dataset (CC=0.083).

| Name | Start | Alignment | End | Score | E-value |
| --- | --- | --- | --- | --- | --- |
| seq_1 | -269 | CGTGCTTTGCGGCGGGCCG | -251 | 16.4996 | 1.27E-08 |
| seq_2 | -156 | CGAGCTGTGCTGCTCGCGG | -138 | 15.8409 | 3.27E-08 |
| seq_3 | -380 | CGAATTTCGCGGCACGACG | -362 | 21.0044 | 2.43E-12 |
| seq_4 | -387 | CTCACTACGCTGCACGAGG | -369 | 17.5515 | 2.58E-09 |
| seq_5 | -245 | CTTGGTGGGCGGACCGAGG | -227 | 14.8335 | 1.25E-07 |

Note: Alignment without positional information demonstrated poor performance (CC=–0.019), implying virtually no overlap with the known binding sites (data not shown).

Supplementary Table 7. Summary of truncation effect on TSS Tompa dataset.

| Data  Subset | [-2000, 0] | | [-1000, 0] | | [-500, 0] | | [-250, 0] | |
| --- | --- | --- | --- | --- | --- | --- | --- | --- |
|  | Without  positional  info | With  positional  info | Without  positional  info | With  positional  info | Without  positional  info | With  positional  info | Without  positional  info | With  positional  info |
| hm01r | -0.012 | -0.007 | -0.011 | 0.005 | 0.014 | 0.148 | -0.007 | -0.007 |
| hm02r | -0.009 | -0.007 | -0.015 | 0.014 | 0.011 | -0.003 | 0.011 | 0.035 |
| hm03r | -0.037 | 0.386 | 0.402 | 0.160 | 0.403 | 0.145 | 0.402 | 0.399 |
| hm04r | -0.008 | -0.005 | 0.054 | -0.005 | 0.131 | 0.197 | 0.123 | 0.197 |
| hm05r | -0.031 | -0.019 | 0.001 | -0.019 | 0.142 | 0.142 | 0.142 | 0.142 |
| hm06r | -0.014 | 0.156 | 0.282 | 0.156 | 0.282 | 0.282 | 0.156 | 0.156 |
| hm07r | -0.015 | -0.015 | -0.012 | -0.008 | -0.007 | -0.010 | 0.238 | 0.238 |
| hm08r | -0.012 | 0.574 | -0.007 | 0.573 | -0.007 | 0.574 | 0.574 | 0.573 |
| hm09r | -0.011 | 0.358 | 0.090 | 0.118 | 0.290 | 0.125 | 0.126 | 0.126 |
| hm10r | -0.019 | 0.083 | 0.083 | 0.083 | 0.083 | 0.083 | 0.087 | 0.087 |
| hm11r | -0.028 | -0.012 | 0.031 | 0.026 | 0.087 | 0.071 | 0.180 | 0.180 |
| hm13r | -0.015 | -0.016 | 0.292 | -0.011 | -0.012 | -0.008 | -0.010 | -0.010 |
| hm14r | 0.204 | -0.018 | 0.204 | 0.145 | -0.020 | -0.020 | -0.012 | -0.012 |
| hm15r | -0.011 | -0.012 | -0.012 | -0.012 | -0.012 | -0.012 | -0.012 | -0.012 |
| hm16r | -0.011 | -0.006 | -0.010 | -0.006 | -0.006 | -0.006 | 0.075 | 0.075 |
| hm17r | -0.015 | -0.012 | 0.213 | 0.195 | 0.213 | 0.196 | 0.205 | 0.205 |
| hm18r | -0.018 | 0.094 | 0.091 | 0.083 | -0.011 | -0.011 | -0.009 | -0.009 |
| hm19r | -0.010 | -0.007 | -0.009 | 0.038 | 0.040 | 0.040 | -0.012 | -0.012 |
| hm20r | -0.026 | 0.046 | -0.019 | 0.159 | 0.096 | 0.191 | 0.084 | 0.094 |
| hm21r | 0.401 | 0.384 | 0.401 | 0.327 | 0.401 | 0.401 | 0.193 | 0.193 |
| hm22r | -0.020 | -0.020 | -0.020 | -0.022 | -0.021 | -0.021 | -0.013 | -0.010 |
| hm24r | -0.016 | -0.010 | -0.013 | 0.048 | 0.108 | 0.165 | 0.109 | 0.109 |
| hm26r | -0.016 | 0.099 | -0.011 | 0.098 | 0.043 | 0.053 | 0.060 | 0.065 |
| CCC | -0.008 | 0.101 | 0.086 | 0.098 | 0.125 | 0.133 | 0.139 | 0.139 |

Supplementary Table 8. Summary of truncation effect on TRANSFAC dataset.

| Data  Subset | Transcription  factor | [-2000, 0] | | [-1000, 0] | | [-500, 0] | | [-250, 0] | |
| --- | --- | --- | --- | --- | --- | --- | --- | --- | --- |
|  |  | Without  positional  info | With  positional  info | Without  positional  info | With  positional  info | Without  positional  info | With  positional  info | Without  positional  info | With  positional  info |
| hm01 | AP-1 | -0.015 | -0.006 | -0.002 | -0.006 | 0.116 | 0.207 | 0.020 | 0.063 |
| hm02 | AP-2 | -0.019 | -0.017 | -0.017 | -0.017 | -0.012 | 0.114 | 0.114 | 0.114 |
| hm03 | AP-2alphaA | -0.015 | -0.008 | 0.031 | -0.008 | 0.066 | 0.068 | 0.028 | 0.043 |
| hm04 | ATF-1 | 0.133 | 0.096 | 0.133 | 0.088 | 0.096 | 0.096 | 0.096 | 0.096 |
| hm05 | ATF-2-xbb4 | 0.017 | 0.337 | 0.083 | 0.356 | 0.356 | 0.356 | 0.356 | 0.337 |
| hm06 | AhR | 0.063 | 0.087 | 0.203 | 0.215 | -0.013 | -0.012 | -0.010 | -0.010 |
| hm07 | BTEB3 | -0.034 | -0.019 | -0.017 | -0.017 | -0.021 | 0.035 | 0.035 | 0.035 |
| hm08 | C/EBPalpha | -0.019 | 0.017 | 0.033 | 0.188 | 0.291 | 0.188 | 0.274 | 0.251 |
| hm09 | C/EBPbeta | -0.020 | -0.007 | 0.077 | -0.008 | -0.010 | 0.082 | 0.007 | 0.007 |
| hm10 | CACCC-binding | -0.009 | -0.011 | -0.011 | -0.011 | 0.155 | 0.155 | 0.571 | 0.583 |
| hm11 | CD28RC | -0.012 | -0.012 | -0.011 | -0.011 | -0.012 | 0.244 | 0.244 | 0.244 |
| hm12 | COUP-TF1 | -0.018 | -0.009 | -0.014 | 0.185 | 0.061 | 0.185 | 0.305 | 0.286 |
| hm13 | COUP-TF2 | -0.012 | 0.263 | 0.060 | 0.263 | 0.231 | 0.253 | 0.239 | 0.253 |
| hm14 | CREB | -0.014 | 0.200 | -0.007 | 0.200 | 0.173 | 0.200 | 0.215 | 0.220 |
| hm15 | CTF | -0.016 | 0.019 | -0.014 | 0.019 | 0.024 | 0.020 | -0.015 | -0.015 |
| hm16 | Copeb | -0.021 | -0.021 | -0.020 | -0.020 | -0.015 | -0.015 | -0.019 | -0.019 |
| hm17 | E12 | -0.010 | -0.015 | -0.015 | -0.015 | -0.017 | -0.015 | -0.017 | -0.015 |
| hm18 | E2F | -0.023 | 0.447 | 0.447 | 0.447 | 0.447 | 0.447 | 0.447 | 0.447 |
| hm19 | E2F-1 | -0.016 | -0.012 | 0.038 | 0.147 | 0.187 | 0.072 | 0.147 | 0.072 |
| hm20 | E47 | -0.016 | -0.016 | -0.016 | -0.016 | 0.027 | 0.027 | -0.018 | -0.018 |
| hm21 | EBF | -0.058 | 0.067 | -0.017 | 0.040 | 0.052 | 0.081 | -0.023 | 0.022 |
| hm22 | ELF-1 | -0.029 | -0.017 | -0.012 | -0.018 | 0.229 | 0.229 | -0.011 | -0.011 |
| hm23 | ER-alpha | -0.014 | -0.008 | -0.011 | -0.008 | -0.011 | -0.008 | -0.005 | -0.009 |
| hm24 | ER-alpha-L | -0.027 | -0.017 | 0.028 | 0.017 | -0.015 | 0.003 | -0.012 | -0.018 |
| hm25 | Egr-1 | -0.002 | -0.008 | -0.011 | -0.006 | 0.029 | -0.008 | 0.101 | 0.046 |
| hm26 | FOXF1 | -0.016 | -0.011 | -0.009 | -0.009 | -0.007 | -0.007 | -0.009 | -0.009 |
| hm27 | FOXO1 | -0.032 | -0.019 | 0.054 | -0.014 | -0.020 | 0.130 | 0.130 | 0.130 |
| hm28 | GATA-1 | -0.020 | 0.000 | 0.009 | 0.009 | -0.013 | -0.013 | 0.121 | 0.000 |
| hm29 | GATA-1 | -0.015 | -0.013 | -0.012 | -0.013 | -0.010 | -0.008 | -0.008 | -0.008 |
| hm30 | GATA-3 | -0.033 | -0.020 | 0.163 | -0.010 | 0.140 | 0.117 | -0.010 | -0.010 |
| hm31 | GLI1 | -0.061 | -0.058 | 0.022 | 0.016 | -0.022 | -0.022 | -0.018 | -0.018 |
| hm32 | GR | -0.017 | -0.008 | -0.014 | -0.012 | -0.012 | -0.012 | -0.011 | -0.010 |
| hm33 | GR-alpha | 0.021 | -0.016 | 0.057 | 0.086 | 0.057 | -0.009 | 0.072 | 0.072 |
| hm34 | HIF-1 | -0.017 | -0.018 | -0.018 | -0.017 | 0.029 | -0.016 | 0.122 | -0.008 |
| hm35 | HMG | -0.026 | -0.019 | 0.076 | -0.019 | 0.042 | -0.012 | -0.011 | -0.012 |
| hm36 | HNF-1alpha | -0.018 | -0.018 | 0.060 | -0.008 | -0.012 | -0.012 | 0.197 | 0.093 |
| hm37 | HNF-1alpha-A | -0.010 | -0.006 | -0.010 | -0.008 | 0.085 | 0.088 | 0.157 | 0.157 |
| hm38 | HNF-1beta-A | -0.030 | -0.024 | 0.185 | 0.439 | 0.439 | 0.439 | 0.439 | 0.439 |
| hm39 | HNF-3alpha | -0.013 | -0.010 | -0.014 | -0.014 | -0.013 | -0.012 | 0.144 | 0.144 |
| hm40 | HNF-4 | -0.024 | -0.015 | -0.016 | -0.012 | 0.005 | 0.056 | 0.069 | 0.026 |
| hm41 | HNF-4alpha | -0.023 | -0.016 | 0.131 | 0.047 | 0.057 | 0.047 | 0.134 | -0.007 |
| hm42 | IPF1 | -0.016 | 0.606 | 0.395 | 0.640 | 0.606 | 0.640 | 0.092 | 0.092 |
| hm43 | IRF-1 | -0.019 | -0.016 | 0.265 | -0.016 | 0.134 | 0.234 | 0.062 | 0.117 |
| hm44 | IRF-8 | -0.020 | -0.020 | -0.010 | -0.009 | 0.200 | 0.200 | -0.008 | 0.287 |
| hm45 | LEF-1 | -0.019 | -0.019 | -0.018 | -0.014 | 0.024 | 0.011 | -0.011 | -0.011 |
| hm46 | MAZ | -0.026 | -0.027 | -0.021 | -0.015 | -0.012 | -0.012 | -0.012 | -0.012 |
| hm47 | MITF | 0.028 | 0.132 | 0.096 | 0.120 | 0.144 | 0.235 | -0.010 | 0.235 |
| hm48 | MZF1B-C | -0.011 | -0.011 | -0.012 | -0.011 | -0.010 | -0.009 | -0.008 | -0.008 |
| hm49 | NF-1 | -0.020 | 0.053 | -0.016 | 0.053 | -0.010 | 0.053 | 0.103 | 0.123 |
| hm50 | NF-1/L | -0.009 | -0.009 | -0.009 | -0.009 | -0.005 | -0.005 | -0.005 | -0.005 |
| hm51 | NF-AT1 | 0.023 | 0.023 | 0.022 | -0.008 | 0.059 | -0.008 | 0.047 | 0.047 |
| hm52 | NF-GMa | -0.010 | 0.583 | 0.583 | 0.583 | 0.583 | 0.583 | 0.583 | 0.583 |
| hm53 | NF-Y | -0.017 | -0.007 | 0.118 | -0.007 | -0.010 | 0.000 | 0.070 | 0.070 |
| hm54 | NF-YA | -0.012 | 0.124 | 0.029 | -0.013 | -0.011 | -0.011 | 0.177 | 0.178 |
| hm55 | NF-kappaB | -0.028 | -0.015 | -0.013 | 0.025 | 0.159 | 0.237 | -0.009 | -0.007 |
| hm56 | NRF-1 | 0.225 | 0.414 | 0.524 | 0.540 | -0.013 | -0.013 | 0.132 | 0.132 |
| hm57 | POU2F1 | -0.013 | -0.009 | 0.055 | 0.088 | -0.010 | -0.008 | 0.088 | 0.088 |
| hm58 | Pit-1A | 0.059 | 0.074 | 0.059 | 0.075 | 0.102 | 0.120 | -0.027 | -0.027 |
| hm59 | RAR-beta | -0.043 | 0.240 | 0.240 | 0.240 | 0.240 | 0.240 | 0.240 | 0.240 |
| hm60 | REST | -0.013 | -0.013 | 0.091 | 0.091 | 0.083 | 0.057 | 0.083 | 0.083 |
| hm61 | RFX1 | -0.025 | 0.286 | 0.286 | 0.286 | 0.286 | 0.286 | 0.293 | 0.293 |
| hm62 | SRF | 0.359 | -0.011 | 0.187 | 0.005 | 0.008 | 0.000 | -0.002 | 0.031 |
| hm63 | SRF-L | -0.015 | -0.013 | -0.013 | -0.013 | -0.013 | -0.013 | 0.069 | 0.069 |
| hm64 | STAT1 | -0.014 | -0.007 | -0.008 | -0.008 | -0.011 | -0.007 | -0.013 | -0.013 |
| hm65 | STAT4 | -0.021 | -0.021 | -0.018 | -0.019 | -0.018 | -0.018 | -0.018 | -0.018 |
| hm66 | Smad3 | -0.015 | -0.009 | -0.013 | -0.011 | -0.012 | 0.095 | 0.099 | 0.099 |
| hm67 | Sp1 | -0.021 | 0.045 | 0.072 | 0.102 | 0.084 | 0.068 | 0.137 | 0.060 |
| hm68 | TCF-4 | -0.019 | -0.019 | -0.019 | -0.019 | -0.013 | -0.013 | -0.015 | -0.015 |
| hm69 | USF1 | -0.019 | 0.009 | 0.153 | 0.354 | 0.045 | 0.354 | 0.185 | 0.354 |
| hm70 | VDR | -0.013 | -0.009 | -0.010 | 0.157 | 0.157 | -0.012 | -0.012 | -0.011 |
| hm71 | WT1 | -0.023 | -0.015 | -0.019 | -0.015 | -0.015 | -0.015 | -0.015 | -0.015 |
| hm72 | YY1 | -0.012 | -0.006 | -0.008 | -0.007 | 0.260 | -0.006 | -0.006 | -0.006 |
| hm73 | c-Ets-1 | -0.019 | -0.016 | 0.012 | -0.008 | -0.011 | -0.008 | -0.011 | -0.010 |
| hm74 | c-Ets-2 | -0.015 | -0.016 | -0.010 | -0.011 | 0.037 | 0.009 | 0.037 | 0.009 |
| hm75 | c-Fos | -0.016 | -0.013 | -0.012 | -0.005 | 0.032 | 0.074 | 0.074 | 0.074 |
| hm76 | c-Jun | -0.009 | -0.006 | -0.009 | -0.007 | 0.043 | -0.008 | 0.135 | 0.131 |
| hm77 | c-Myb-isoform1 | 0.058 | 0.025 | 0.025 | 0.025 | 0.025 | 0.025 | -0.036 | -0.036 |
| hm78 | c-Myc | 0.159 | 0.157 | 0.159 | 0.051 | 0.144 | 0.162 | 0.095 | 0.100 |
| hm79 | c-Rel | -0.014 | -0.012 | -0.012 | -0.012 | -0.008 | -0.009 | -0.008 | -0.008 |
| hm80 | p50 | -0.018 | -0.013 | -0.016 | -0.012 | 0.119 | 0.024 | 0.594 | 0.594 |
| hm81 | p53 | -0.021 | -0.015 | 0.103 | -0.007 | -0.009 | 0.077 | 0.065 | 0.065 |
| hm82 | p53-isoform-1 | -0.019 | 0.023 | -0.015 | 0.045 | -0.016 | -0.015 | -0.014 | -0.014 |
| CCC |  | -0.009 | 0.027 | 0.050 | 0.066 | 0.077 | 0.078 | 0.094 | 0.076 |
